# Versatile Ice Microneedles for Transdermal Delivery of Diverse Actives

**DOI:** 10.1002/advs.202101210

**Published:** 2021-07-03

**Authors:** Xiaoxuan Zhang, Xiao Fu, Guopu Chen, Yuetong Wang, Yuanjin Zhao

**Affiliations:** ^1^ Department of Rheumatology and Immunology Institute of Translational Medicine The Affiliated Drum Tower Hospital of Nanjing University Medical School Nanjing 210002 China; ^2^ State Key Laboratory of Bioelectronics School of Biological Science and Medical Engineering Southeast University Nanjing 210096 China

**Keywords:** freeze, hydrogel, ice microneedles, transdermal delivery

## Abstract

Microneedles are regarded as an emerging and promising transdermal drug delivery strategy. Great efforts are devoted to getting rid of their material restrictions and imparting them with abilities to carry various drugs. Here, inspired by ice formation in nature and based on characteristics of different frozen materials, the authors present novel ice microneedles made from versatile soft materials using a simple freezing template‐based fabrication stratagem for effective transdermal delivery of diverse actives. Their strategy can convert microneedles with almost all water‐containing components from softness into hardness for guaranteeing satisfactory penetration, thus removing their material component limitations. As all fabrication procedures are mild and actives can maintain activity during these processes, the ice microneedles can carry and deliver various actives from small molecules and macromolecules to even living organisms. They have demonstrated that these ice microneedles can easily penetrate mouse and swine skins using a microneedle injector, with their active‐carried tips left inside after their ice base melts. Thus, by loading heparin, erythropoietin, or biosafe *Bacillus subtilis* (*B. subtilis*) inside the ice microneedles to treat mouse models, the practical values of these microneedles are well displayed, indicating their bright prospects in universal drug delivery systems.

## Graphical abstract

Ice microneedles are fabricated by a mild and easy‐to‐operate freezing‐template‐replication stratagem and can convert water‐containing components from softness into hardness. Thus, these ice microneedles can be made from versatile soft materials, removing the material limitations; also, they can maintain the activity of carried actives including small molecules, macromolecules, living organisms, etc., ensuring transdermal delivery of diverse actives.

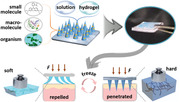

## 1 Introduction

Transdermal drug delivery is one of the major drug delivery strategies, which can avoid gastrointestinal tract interferences, extend effecting time, reduce side effects, and simplify administration processes.^[^ ^1^, ^2^, ^3^, ^4^, ^5^ ^]^ For effective transdermal drug delivery, numerous methods have been put forward and multiple devices have been developed, such as ointments,^[^ ^6^, ^7^, ^8^, ^9^ ^]^ plasters,^[^ ^10^, ^11^, ^12^, ^13^ ^]^ patches,^[^ ^14^, ^15^, ^16^, ^17^ ^]^ microneedles,^[^ ^18^, ^19^, ^20^ ^]^ etc. Among them, microneedles could penetrate the epidermis without touching blood capillaries and nerve endings, thus enhancing drug absorption in a painless, minimal‐invasive way.^[^ ^21^, ^22^ ^]^ Benefitting from these outstanding properties, microneedles have been widely applied to the release of various actives, including small molecule drugs, proteins, and vaccines.^[^ ^23^, ^24^, ^25^, ^26^, ^27^, ^28^ ^]^ However, to achieve successful penetration, the materials of microneedles should satisfy the strength and hardness requirements, which impose great limitations on the choices of their components and thus restrict the applications of microneedles. In addition, many actives are very demanding on the surrounding environments and each has different requirements for loading conditions, while there are no such microneedles that are broadly applicable to the carriage and delivery of almost all actives, including the vulnerable or cell‐related ones. Therefore, it is still highly anticipated to develop versatile microneedles with advanced functions for more generalized applications.

**Figure 1:**
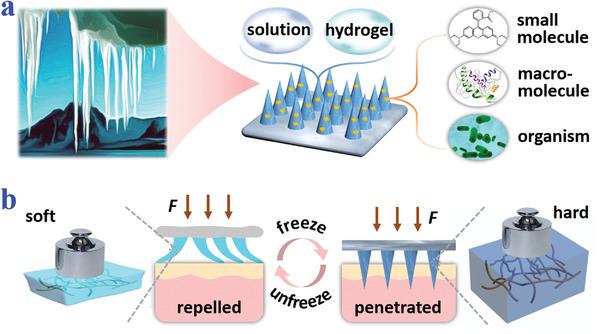
Schematic illustrations of compositions and properties of ice microneedles. a) Ice‐formation‐inspired ice microneedles can be made from soft materials such as solutions and hydrogels, and are able to carry diverse actives including small molecules, macromolecules, and living organisms. b) Scheme of transformation from softness to hardness realized by freezing processes.

Here, inspired by ice formation in nature and based on the characteristics of different frozen materials,^[^ ^29^, ^30^, ^31^, ^32^ ^]^ we present novel ice microneedles with the desired versatile features for all‐purpose transdermal delivery of diverse actives by using a simple freezing template‐based fabrication stratagem, as schemed in **Figure** **1**. Such ice microneedles could get rid of the material limitations and be composed of almost all water‐containing materials (e.g., solutions, temperature curing materials, light curing materials, and ion‐crosslinking curing materials), which were converted from softness into hardness for guaranteeing their satisfactory penetration. In addition, as such freezing process did no harm to the activity of the carried bioactives, these ice microneedles could successfully deliver small molecules such as Rhodamine B (RhB), proteins such as bovine serum albumin (BSA), and even live organisms such as probiotic bacteria. It was demonstrated that the tips of the ice microneedles could penetrate different animal skins by employing a microneedle injector and be left inside the skin for continuous and complete delivery after the ice base melted. Thus, these ice microneedles were used to carry heparin, erythropoietin, as well as biosafe *Bacillus subtilis* (*B. subtilis*) for anticoagulation, treating anemia or fungal infection mouse models. All these results indicated that the ice microneedles could be ideal candidates for practical and universal transdermal delivery.

## 2 Results and Discussions

**Figure 2:**
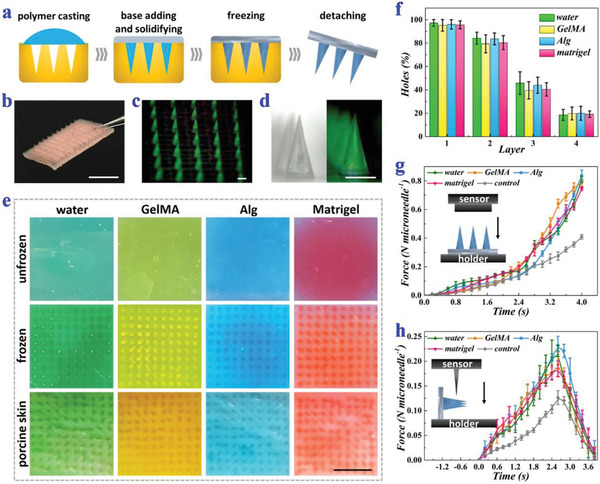
Fabrication, characterization, and mechanical strengths of ice microneedles. a) Schemes of the fabrication process of ice microneedles. b) Digital image of an ice microneedle patch. c) Fluorescence image of an ice microneedle array. d) Bright field and fluorescence field images of a single ice microneedle. e) Digital images of agarose after penetration of unfrozen water microneedles (dyed green), GelMA microneedles (dyed yellow), Alg microneedles (dyed blue), and Matrigel microneedles (dyed red); digital images of agarose after penetration of ice microneedles; digital images of porcine skins after penetration of ice microneedles. f) Penetration efficiency of ice microneedles into different layers of parafilm. The ice microneedles are made from water, GelMA, Alg, and Matrigel (*n* = 5 for each group). g) Compressive forces of four kinds of ice microneedles and PEGDA control ones (*n* = 3 for each group). h) Shear forces of four kinds of ice microneedles and PEGDA control ones (*n* = 3 for each group). Scale bars: 0.5 cm in (b,e) and 500 µm in (c,d).

In a typical experiment, the ice microneedles were fabricated by introducing a freezing step to the traditional template replication approach, as schemed in **Figure** **2**a. Specifically, the prepolymer of microneedle materials was first applied and filled into the cavities of a negative template by vacuum treatment. After polymerization, a piece of soggy gauze was added to cover the negative template and they were frozen together. Finally, by detaching them from the negative template, the ice microneedles could be obtained and they should be preserved below zero degree. The resultant ice microneedles had a 10 × 10 array, whose tips were about 880 µm in height and 500 µm in diameter (Figure 2b–d). Besides, it was found that both the heights and diameters of the tips were distributed over a relatively small range, indicating the satisfactory uniformity (Figure S1, Supporting Information).

The hardness of almost all water‐containing materials was distinctively enhanced in the frozen state. To demonstrate this, different types of materials including water, temperature curing material (Matrigel), light curing material (methacrylate gelatin, GelMA), and ion‐crosslinking curing material (alginate, Alg) were chosen as representative microneedle materials and their strengths before and after freezing were compared. It was found that without freezing, GelMA and Alg could not be completely demolded, and water and Matrigel could not even form a microneedle shape (Figure S2, Supporting Information). On the contrary, all materials could be easily demolded with intact microneedle shape after being frozen. The penetration abilities of microneedles made from these materials were further tested. Results showed that unfrozen microneedles hardly made any holes in agarose, while frozen microneedles could successfully pierce both agarose and porcine skin (Figure 2e). Besides, ice microneedles composed of all the four materials achieved the penetration efficiency of more than 90%, indicating their ideal penetration capacity (Figure S3, Supporting Information).

To further investigate the mechanical strengths of ice microneedles, other qualitative and quantitative experiments were conducted. As demonstrated by the microneedle‐created holes in different layers of a wad of parafilm, the ice microneedles had the ability to penetrate the first parafilm layer with the ratio nearly 100% and could touch as deep as the forth layer (Figure 2f). Besides, the forces of ice microneedles under compression or shear were recorded and evaluated using a force sensing machine. Noteworthily, microneedles made of 80% poly(ethylene glycol) diacrylate (PEGDA), a commonly used microneedle material, were tested as the control group. It was found that the maximum compressive force which each ice microneedle could bear was up to 0.8 N on average, which was almost twice as much as that of the control group and far exceeded the requirements for penetrating the skin (Figure 2g). As for the maximum shear force, it was about 0.2 N per needle for the ice microneedles, which was also significantly higher than the control group (Figure 2h). In addition, the mechanical strengths of ice microneedles were found to be affected by the freezing time and freezing temperature. When the freezing time was short, the ice microneedles were not completely frozen, thus their strengths and hardness were relatively low (Figure S4, Supporting Information). As the freezing time extended, their strengths increased and reached the peak in about 1 day. It could also be observed that mechanical strengths of ice microneedles were higher when frozen at −70 °C than when frozen at −20 and −15 °C (Figure S5, Supporting Information).

**Figure 3:**
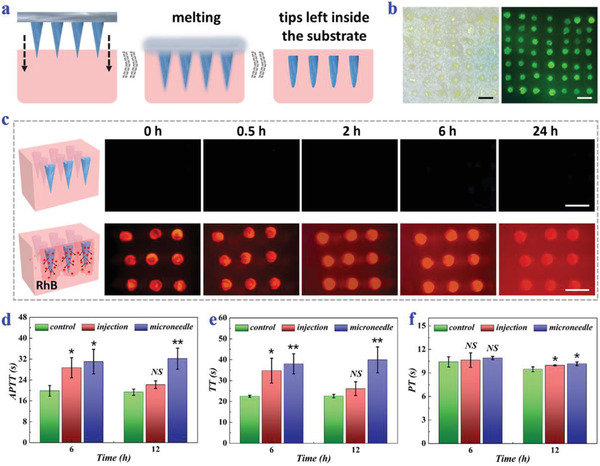
Penetration, separation, and small molecule release abilities of ice microneedles. a) Schematic illustrations showing the penetration and separation of ice microneedles. b) Bright‐field and fluorescence images of microneedle tips left in the porcine skin. c) Fluorescence images of unloaded ice microneedles (above) and RhB‐loaded ones (below) in agarose at different time points. d–f) APTT (d), TT (e), and PT (f) of normal mice, mice receiving tail vein injection of heparin, and mice receiving heparin‐loaded ice microneedles for 6 and 12 h (*n* = 3 for each group; Student's *t*‐test was conducted for comparison; * *p* < 0.05, ** *p* < 0.01, NS: nonsignificant). All scale bars: 1 mm.

After penetrating the substrate, the base of ice microneedles would melt, leaving the tips in the substrate, as schemed in **Figure** **3**a. The ice microneedles were first applied to agarose, which was usually used as the skin model. Both the top view images and the side view images evidenced the fine penetration and separation abilities of these ice microneedles (Figure S6a,b, Supporting Information). The penetration depths were also measured and most were found to range from 720 µm to 860 µm, as shown in Figure S6c, Supporting Information. In addition, the ice microneedles were further employed to insert into fresh porcine skin. Similarly, they could successfully penetrate the porcine skin and leave their tips inside after melting (Figure 3b).

Benefitting from the above attributes, various types of drugs could be encapsulated inside the microneedle tips for sustainable release. To visualize the release profile of small molecules, RhB, a model small‐molecule drug, was loaded in the ice microneedles. These RhB‐loaded ice microneedles were then applied to agarose and fluorescence images were taken at specific time points. The increasing fluorescence intensity of the nearby agarose demonstrated that RhB could diffuse from the ice microneedles in a continuous way (Figure 3c). Besides, their in‐vivo small molecule release ability and therapeutic effects were also evaluated by applying heparin‐loaded ice microneedles to BALB/c mice. It was found that the activated partial thromboplastin time (APTT) and thrombin time (TT) of mice receiving heparin‐loaded ice microneedles or heparin injection had a significant increase, and the prothrombin time (PT) of both groups showed no obvious difference (Figure 3d–f). Compared to heparin injection, which was only effective in a short term, ice microneedles could remain good anticoagulant effects up to 12 h. Such results indicated heparin could be sustainably released into the body via these ice microneedles and function normally.

Noteworthily, during the process of applying ice microneedles to the mice, a microneedle injector was employed to assist the microneedle penetration for better and easier operation (Figures S7 and S8, Supporting Information). To be specific, ice microneedles whose base was fixed to the injector would puncture the skin in an instant with the help of the forces created by spring extension inside the injector. The injector kept on the skin until the ice melted and the tips detached. For visually demonstrating the skin piercing and displaying the penetration depths, the ice‐microneedle‐applied mouse skin was sampled and stained. The layer‐by‐layer hematoxylin and eosin staining (H&E staining) images showed that the ice microneedles successfully penetrated the mouse skin and the penetration depth was more than 400 µm (Figure S9, Supporting Information).

**Figure 4:**
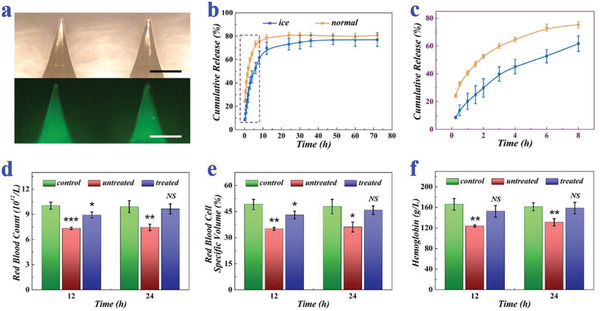
Release and therapeutic behaviors of macromolecular‐drug‐loaded ice microneedles. a) Optical and fluorescence images of FITC‐BSA‐loaded ice microneedles. b,c) Release profiles in PBS buffer solution of both FITC‐BSA‐loaded ice microneedles and FITC‐BSA‐loaded unfrozen microneedles (*n* = 6 for each group). d–f) RBC (d), HCT (e), and HGB (f) of normal mice, untreated anemia mice, and treated anemia mice after receiving erythropoietin‐loaded ice microneedles for 12 and 24 h (*n* = 3 for each group; Student's *t*‐test was conducted for comparison; * *p* < 0.05, ** *p* < 0.01, *** *p* < 0.001, NS: nonsignificant). Both scale bars: 500 µm.

To investigate their ability of releasing macromolecular drugs, ice microneedles loaded with fluorescein isothiocyanate‐labelled BSA (FITC‐BSA) were prepared for in‐vitro release tests (**Figure** **4**a). Results showed that the initial release rates of ice microneedles were slightly lower than their unfrozen counterparts, probably because the ice microneedles would first go through a thawing process (Figure 4b,c). Whereas, their final cumulative release amounts were very close to each other, which were about 80%. In addition, erythropoietin, a glycoprotein hormone that could stimulate erythrocyte production, was encapsulated in the ice microneedles, which were then applied to anemia mice. For the establishment of the renal anemia mouse model, mice were fed with adenine, which could reduce renal lesions, inhibit the secretion of erythropoietin, and lead to hematopoietic dysfunction. Thus, those untreated anemia mice displayed a sharp declination in red blood count (RBC), red blood cell specific volume (HCT), and hemoglobin (HGB) compared to normal mice, as shown in Figure 4d–f. In contrast, after receiving erythropoietin‐loaded ice microneedles, all of RBC, HCT, and HGB gradually increased to the normal level, indicating that erythropoietin could keep active in ice microneedles and be released in a stepwise way.

**Figure 5:**
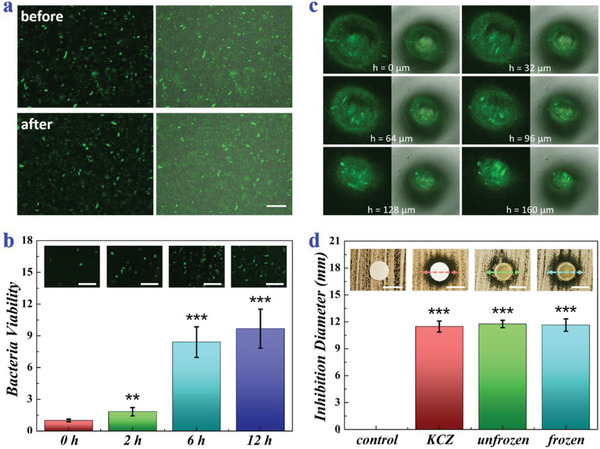
*B. subtilis* viability and functions after freezing. a) Fluorescence field and superposed field images and of SYTO‐dyed *B. subtilis* in hydrogel blocks before and after freezing. b) Quantitative analysis and corresponding confocal microscopy images of *B. subtilis* in hydrogel blocks after thawing for 0, 2, 6, and 12 h (*n* = 4 for each group; Student's *t*‐test was conducted for comparison; ** *p* < 0.01, *** *p* < 0.001). c) Fluorescence field and superposed field images of SYTO‐dyed *B. subtilis* in the microneedles. The images differ in the height of *z* axis. d) Quantitative analysis and corresponding optical images of the inhibition rings against *C. albicans* (*n* = 5 for each group; Student's *t*‐test was conducted for comparison; *** *p* < 0.001). Scale bars: 20 µm in (a b) and 6 mm in (d).

Furthermore, it was realizable to encapsulate living organisms such as cells and microbes in the ice microneedles. To demonstrate this, *B. subtilis*, one of the beneficial bacteria which naturally located on human skins and intestinal tracts, was chosen and loaded in the ice microneedles. For evaluating the effects of freezing on *B. subtilis* viability, the hydrogel blocks containing *B. subtilis* with or without freezing were dyed with SYTO. It was observed that the relative contents of live bacteria in frozen and unfrozen hydrogels were basically the same, indicating that freezing did no harm to *B. subtilis*, as shown in **Figure** **5**a and Figure S10, Supporting Information. Notably, *B. subtilis* could continue to divide and proliferate in the hydrogel after thawing, with the growth trends originally increasing exponentially and then slowing down until saturation due to the limitations of nutrients and space (Figure 5b). The bacteria together with the hydrogel were filled into the negative template to fabricate ice microneedles with the aforementioned approach. Judging from the confocal microscopy images, the bacteria could well distribute in the ice microneedles (Figure 5c and Figure S11, Supporting Information).

Since *B. subtilis* could secrete antimicrobial agents and compete with other colonies for nutrients and space, it had broad‐spectrum antimicrobial properties against both bacteria and fungi, providing a new idea to treat intractable recurrent fungal infections. Thus, our *B. subtilis*‐loaded ice microneedles (B‐MNs) might be a promising candidate for fungal infection treatments. To verify this, inhibition ring tests were carried out. During the tests, pure paper filter discs, paper filter discs carrying 20 µg ketoconazole (KCZ, an antifungal drug), unfrozen *B. subtilis*‐loaded hydrogel discs, and frozen *B. subtilis*‐loaded hydrogel discs were placed on petri dishes spread with a lawn of *Candida albicans* (*C. albicans*), respectively. After incubation for 24 h at 37 °C, fungistatic zones could be clearly seen in the KCZ group, the unfrozen *B. subtilis* group, as well as the frozen *B. subtilis* group, as shown in Figure 5d. It was worth mentioning that there were negligible differences in the inhibition ring lengths of the three groups, indicating that the antifungal activity of *B. subtilis* would not be impeded by freezing and that the ice microneedles could have a comparable antifungal effect to KCZ.

**Figure 6:**
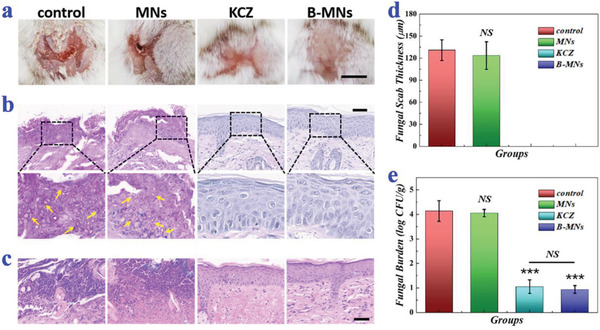
In‐vivo antifungal performances of *B. subtilis*‐loaded ice microneedles. a) Representative photos of back skins of the mice in the control group, the MNs group, the KCZ group, and the B‐MNs group on day 13. b) PAS staining of mouse back skins in different groups on day 13. The arrows pointed to the pseudohyphae of *C. albicans*. c) H&E staining of mouse back skins in different groups on day 13. d) Quantitative analysis of the fungal scab thicknesses of the mice (*n* = 4 for each group; Student's *t*‐test was conducted for comparison; NS: nonsignificant). e) Quantitative analysis of fungal burden of the infected skin tissues (*n* = 6 for each group; Student's *t*‐test was conducted for comparison; *** *p* < 0.001, NS: nonsignificant). Scale bars: 0.5 cm in (a) and 50 µm in (b,c).

Besides, in‐vivo antifungal abilities of B‐MNs were also evaluated and a cutaneous fungal infection model was established in mice. Before fungal infection, the mice were first injected with cyclophosphamide and hydrocortisone acetate for immunosuppression (Figure S12, Supporting Information). To induce fungal infections, their back skins were then rubbed with blades and the injured skins were smeared with *C. albicans* suspension. After that, the mice were randomly divided into four groups and received no treatments (control group), bare ice microneedle (MNs group), KCZ cream (KCZ group), and B‐MNs (B‐MNs group) for 12 days, respectively. During the treatments, the changes of the mouse back skins were recorded. Results showed that fungal infections in B‐MNs group were obviously reduced after the first day of treatment (Figure S13, Supporting Information). After 12‐day treatment, in contrast to the still severe infections of control group and MNs group, no serious fungal infection could be seen for the KCZ group and the B‐MNs group and hair began to regrow on the past infected areas (**Figure** **6**a).

For detailed assessment of the skin infection condition, histopathological analysis was conducted. Judging from the periodic Acid‐Schiff staining (PAS staining), a large number of pseudohyphae of *C. albicans* could be seen in the control group and the MNs group (Figure 6b). On the contrary, almost no pseudohyphae were found in the KCZ group and the B‐MNs group, suggesting the satisfactory antifungal effects. To show the skin recovery and inflammation states, the skin samples were also stained with hematoxylin and eosin. Results showed that a quantity of mononuclear and polymorphonuclear cells infiltrated into the epidermis in the control group and the MNs group; while few inflammatory cells could be observed in the KCZ group and the B‐MNs group, indicating that there was little inflammatory response and that the skins almost recovered to normal (Figure 6c). In addition, zero thickness of the fungal scab and the extremely low fungal burden of the mice in B‐MNs group among all the other groups further demonstrated the curative effects of B‐MNs, which were comparative or even more effective than commercial drugs (Figure 6d,e).

As the microneedle tips would be left inside the body, the safety of *B. subtilis* should be paid special attention. For this purpose, a high concentration of *B. subtilis* suspensions was subcutaneously injected to the back of the mice and the changes of their body weights were measured. Slight weight loss of these mice were observed in the initial 2 days, while their body weights steadily gained since the third day, as shown in Figure S14, Supporting Information. In addition, the back skins near the injection were sampled and stained for more detailed analysis. Although a little inflammatory cell invasion was found in the epidermis on day 1 after bacteria injection, few could be seen in the skin samples on day 4 and day 7, indicating the rapid recovery of skin and the minimal infection or inflammation in the long term (Figure S15, Supporting Information). The H&E staining together with the Masson staining also displayed the clear skin layers and the regularly orderly distributed collagens of the mouse skins on day 4 and day 7. The above results strongly evidenced the biosafety and compatibility of the *B. subtilis*.

## 3 Conclusion

In summary, inspired by natural ice formation, we generated versatile ice microneedles using a freezing template‐based fabrication approach to get rid of material limitations and realize all‐purpose active delivery. During the fabrication, the microneedles composed of water‐rich materials were frozen before being peeled from the template, so that their structures were maintained and their hardness was distinctively enhanced. It was found that the mechanical strengths of ice microneedles from water, Matrigel, GelMA, and Alg were significantly higher than their unfrozen counterparts and could fulfill the demands of skin penetration. Similar to other biological agents, these ice microneedles should be kept sterile during storage and transport at −20 or −80 °C for a longer time, which was easy to implement. Notably, after being penetrated into the agarose or ex‐vivo skins via an injector, the ice microneedles would gradually melt, with their tips separating from the base and being left inside for continuous release of the carried actives. Additionally, as the freezing process did not destruct the activity of most actives, a wide range of actives could be loaded in and delivered by those ice microneedles, including small molecules, proteins, and live organisms. To be noted, the delivery efficiency of the ice microneedles was determined by their material components and the properties of the carried actives. It has been further demonstrated that the ice microneedles could deliver heparin or erythropoietin for anticoagulation or treating anemic mice. Moreover, ice microneedles loaded with *B. subtilis*, a type of beneficial bacteria with antimicrobial abilities, have shown satisfactory therapeutic effects in treating fungal infections in immunosuppressed mice. All these features indicated the practical values of ice microneedles in transdermal drug delivery, disease treatments, and many other fields.

Compared to traditional fungal infection treatments including topical cream, suppository, oral drug administration, etc., the employment of B‐MNs possesses plenty of advantages. For example, *B. subtilis* can not only excrete various antifungal agents which aim at specific proteins of fungal cells, destroy the cell walls, and kill the fungi, but also repel and inhibit the reproduction of fungi by preempting space and nutrients. Thus, the B‐MN treatment can avoid the occurrence of resistance. Besides, due to the biosafety of B‐MNs, such treatments can reduce side effects. Also, the relapse of fungal infections is hindered using this treatment. In addition to carrying probiotic bacteria, these ice microneedles are promising to load genetically engineered bacteria to treat other types of diseases including metabolic disorders and cancer. By optimizing the freezing steps and materials, stem cells may also be encapsulated inside the ice microneedles. It is believed that the emergency of ice microneedles can provide lots of inspirations in the development of new‐generation microneedles and shines in the translational medicine field.

## 4 Experimental Section

### Materials, Microorganisms, and Animals

Methacrylate gelatin (GelMA, crosslinking degree ≈90%) was provided by Aladdin. Sodium alginate (low viscosity) and agarose were bought from Alfa Aesar. Matrigel was obtained from Corning. 2‐hydroxy‐2‐methylpropiophenone (HMPP), calcium chloride, and rhodamine B (RhB) were purchased from Sigma‐Aldrich. Fresh porcine skins were bought from the market. Heparin, erythropoietin, cyclophosphamide, cyclophosphamide, and ketoconazole (KCZ) were provided by Drum Tower Hospital. FITC‐BSA was from Zhongkechenyu Biotech. SYTO was offered by KeyGEN Biotech. Sabouraud dextrose agar medium (SDA), nutrient agar medium (NA), and Luria–Bertani culture medium (LB) were purchased from Hopebio Co., Ltd. Deionized water (resistivity: 18.2 MΩ cm^−1^) was purified by a Milli‐Q water purification system (Millipore). *B. subtilis* 3610 and *C. albicans* SC5314 were received from BeNa Biotech Co., Ltd. Male BALB/c mice (about 8 weeks and 20 g) were obtained from Drum Tower Hospital. Animals were treated in strict accordance with the Beijing Administration Rule of Animals in China and had received approval from Animal Investigation Ethics Committee of Drum Tower Hospital.

### Fabrication of Ice Microneedles

To fabricate water‐composed ice microneedles, water was first added to a negative template with ordered cavities. After 5‐min vacuum treatment (vacuum degree: −15 psi), 200 µL water could be filled into the cavities and the extra water was removed using a pipette. A piece of soggy gauze was then placed on the cavities, which was frozen at −70 °C together with the template for 24 h. The ice microneedles would be obtained by removing the template and should be stored below 0 °C. To fabricate GelMA‐composed ice microneedles, 10% (w/v) GelMA solution mixed with 1% (v/v) HMPP was added to the template as microneedle materials. Before being frozen, the GelMA‐filled template was exposed to UV irradiation (365 nm) for 30 s. All other steps were similar to those of fabricating water‐composed ice microneedles. To fabricate Alg‐composed ice microneedles, 4% (w/v) sodium alginate was set as the prepolymer solution of microneedle materials, which was solidified by reacting with 10% (w/v) CaCl_2_ for 10 min. Other steps were also similar. For Matrigel‐composed ice microneedles, the only differences were that Matrigel was filled into the template and it was solidified at room temperature. The full pictures of microneedles were taken using a Huawei P30 pro mobile phone. The fluorescence images were acquired using a biological microscope (FSX 100, Olympus). Other optical images were captured by a stereomicroscope (JSZ6S, Jiangnan novel optics) and a matched CCD camera (Oplenic digital camera).

### Agarose, Porcine Skin, and Parafilm Penetration

The agarose, porcine skin, and parafilm were cut into small blocks, respectively. These blocks and an iron‐made mini‐tamp were then placed at 4 °C for precooling. To observe the holes created by microneedles, the microneedles were pressed to the substrates by the mini‐tamp for 5 s and then were removed quickly. By counting the holes in the agarose, the penetration efficiency of different types of microneedles could be calculated. By using multiple layers of parafilm as the substrates, the penetration behaviors of microneedles on different layers could be obtained.

### Compressive and Shear Force Tests

The compressive and shear forces were measured using a mechanical sensing system (ZHIQU Co. Ltd., Guangzhou, China). This system contained an upper movable end, a bottom fixed end, and a force sensor. During the compressive force tests, the ice microneedles were placed on the ice‐bag‐cooled fixed end with their tips facing the movable end. The movable end then gradually approached the ice microneedles with the speed of 0.2 mm s^−1^. The force recording started once the movable end touched the microneedle tips, and stopped after travelling for 4 s. During the shear force tests, the ice microneedles were fastened sideways on the fixed end, and a sharp blade was glued to the movable end. The approaching speed was about 0.1 mm s^−1^. Similarly, the force recording began as soon as the movable ends of the microneedle tips contacted each other, and ended until they departed.

### Application of Ice Microneedles to Mice

The naked skins of the mice first experienced ice compress for over 30 s and were then wiped with 75% (v/v) alcohol for cooling. The base of the ice microneedles was fixed to a microneedle injector beforehand and they were used immediately once taken to room temperature. The tips of ice microneedles would penetrate the mouse skin as soon as the button on the microneedle injector was pressed to release the inside spring and make it extend. It was measured that the force generated by the microneedle injector was about 8 n. The ice melted in less than 1 min, and then the microneedle injector together with the base could be removed, leaving the tips inside the skin.

### Heparin‐Loaded Ice Microneedles for Anticoagulation

The GelMA compound hydrogel containing 4000 U mL^−1^ heparin was set as the microneedle material. The mice were randomly divided into three groups. Each group had three mice at each time point. For the control group, the mice were injected with 200 µL saline via tail vein. For the injection group, 200 µL of heparin sodium solution (200 U mL^−1^) was injected into the mice in the same way. For the microneedle group, the ice microneedles containing heparin (total volume 0.01 mL) were applied to the backs of the mice. Blood was collected from the eyes of the mice and used for blood coagulation tests after 6 and 12 h, respectively.

### In‐Vitro FITC‐BSA Release

Hydrogel blocks with the same compositions as the tips of GelMA‐composed ice microneedles were prepared for in‐vitro FITC‐BSA release tests. FITC‐BSA was encapsulated inside these hydrogel blocks with the initial concentration of 1 mg mL^−1^. These hydrogel blocks were then immersed in PBS buffer solution and kept in a thermomixer (Eppendorf, speed: 300 rpm min^−1^, 37 °C). At specific time points, 100 µL PBS solution was collected in a 96‐well plate, and another 100 µL fresh PBS solution was supplemented. The fluorescence intensity of the collected PBS solution could be read out using a multimode plate reader (excitation wavelength: 495 nm, emission wavelength: 550 nm). The cumulative release amount over time could be finally calculated from the real‐time fluorescence intensity and the standard concentration‐intensity curve of FITC‐BSA.

### Erythropoietin‐Loaded Ice Microneedles for Anemia Treatment

The GelMA compound hydrogel containing 5000 U mL^−1^ erythropoietin was set as the microneedle material. To establish the renal anemia mouse model, the mice were fed with fodder containing 0.75% adenine (300 mg kg^−1^ body weight per day) for consecutive seven weeks. The ice microneedles containing erythropoietin were applied to the backs of the mice for treatment. Blood was collected from the eyes of the mice and used for blood routine tests after 12 and 24 h, respectively. It should be mentioned that there were 3 mice at each time point for the normal control group, untreated anemia group, and microneedle‐treated anemia group, respectively.

### Evaluations on *B. subtilis* Viability

*B. subtilis* was reserved in glycerin‐PBS solution (1:1) mixture at −80 °C and cultured on a NA medium petri dish at 37 °C. One colony was then transferred to a 15 mL centrifuge tube containing LB culture medium mixed with 10% (w/v) GelMA and 1% (v/v) HMPP. To evaluate the bacteria viability in the GelMA compound hydrogel, live bacteria before freezing and 0, 2, 6, and 12 h after recovery were dyed by SYTO and characterized using a laser scanning confocal microscopy (FV3000, Olympus).

### Inhibition Ring Tests

*C. albicans* was maintained in glycerin‐PBS solution (1:1) mixture at −80 °C and resurrected on the SDA medium petri dish for 24 h at room temperature. The conidia were collected, diluted to 10^9^ mL^−1^, and spread on SDA medium petri dishes. Pure paper filter discs, paper filter discs loaded with 20 µg KCZ, unfrozen GelMA compound hydrogel discs loaded with 10^12^ mL^−1^ *B. subtilis*, and frozen GelMA compound hydrogel discs loaded with 10^12^ mL^−1^ *B. subtilis* were placed on the petri dishes, respectively. Each group has five parallels. The diameters of all the discs were 6 mm. The lengths of inhibition rings were measured after incubation for 24 h at 37 °C.

### B. Subtilis‐Encapsulated Ice Microneedles for Fungal Infection Treatment

The GelMA compound hydrogel carrying 10^12^ mL^−1^ *B. subtilis* was set as the microneedle material. The first step of establishing the fungal infection model was to induce immunosuppression. For this purpose, mice were intraperitoneally injected with cyclophosphamide (150 mg kg^−1^ of body weight) at day −3 and day −1 (the infection day as day 0), and subcutaneously injected with hydrocortisone acetate (40 mg kg^−1^ of body weight) at day ‐1. The hair on their backs was then removed and the naked skins were rubbed with blades until the blood oozed. The injured skins were immediately smeared with 10^9^ mL^−1^ *C. albicans* suspension using sterile swabs for three times. The treatments were conducted once a day and lasted from day 1 to day 12. During the treatments, the mice were randomly divided into four groups (four mice in each group): the control group (receiving no treatments), the MNs group (receiving bare ice microneedles without *B. subtilis*), the KCZ group (receiving 25 mg of 2% KCZ cream), and the B‐MNs group (receiving ice microneedles with *B. subtilis*). The everyday changes of mouse back skins were recorded. At day 13, the mice were sacrificed and their infected skin tissues were sampled. Hematoxylin and eosin staining (H&E staining) and PAS staining were conducted for further histological analysis. To calculate skin fungal burden, skin tissues were sliced aseptically and homogenized in PBS. The tissue homogenates were plated in serial dilutions on yeast extract/peptone/dextrose (YPD) agar plates. After incubation at 30 °C for 24 h, colony‐forming units (CFU) were counted.

### Safety Evaluation of *B. subtilis*

To evaluate the safety of *B. subtilis*, 100 µL of suspension solutions containing 10^20^ mL^−1^ *B. subtilis* were subcutaneously injected to the mice. The mice were weighed once a day for 7 days. Their back samples were collected and immersed in neutral formaldehyde at day 1, day 4, and day 7. The samples were then taken out from the neutral formaldehyde, dehydrated, embedded in paraffin, and serially sectioned for H&E analysis and Masson analysis, respectively.

### Statistical Analysis

The data were normalized based on the control group. All the presented data were displayed as mean ± SD. The sample sizes (*n*) were provided in the figure legends. To assess the differences between different groups, Student's *t*‐test was conducted. The difference was regarded to be statistically significant if *p* < 0.05. SPSS 20.0 software was used to perform statistical analysis.

## Conflict of Interest

The authors declare no conflict of interest.

## Supporting information

Supporting Information

## Data Availability

Research data are not shared.
