# Supplementary material for: Versatile Ice Microneedles for Transdermal Delivery of Diverse Actives
Source: Adv Sci (Weinh). 2021 Jul 3;8(17):2101210. doi: 10.1002/advs.202101210 (PMC8425882; doi:10.1002/advs.202101210)
Supplement: Supplementary file 1 — Supporting Information [file ADVS-8-2101210-s001.pdf]

WILEY-VCH

© 2021 Wiley-VCH GmbH

ADVANCED SCIENCE
Open Access

Supporting Information

for *Adv. Sci.*, DOI: 10.1002/advs.202101210

Versatile ice microneedles for transdermal delivery of diverse actives

*Xiaoxuan Zhang, Xiao Fu, Guopu Chen, Yuetong Wang, Yuanjin Zhao**

Supporting Information

Versatile ice microneedles for transdermal delivery of diverse actives

*Xiaoxuan Zhang, Xiao Fu, Guopu Chen, Yuetong Wang, Yuanjin Zhao**

Supporting Figures

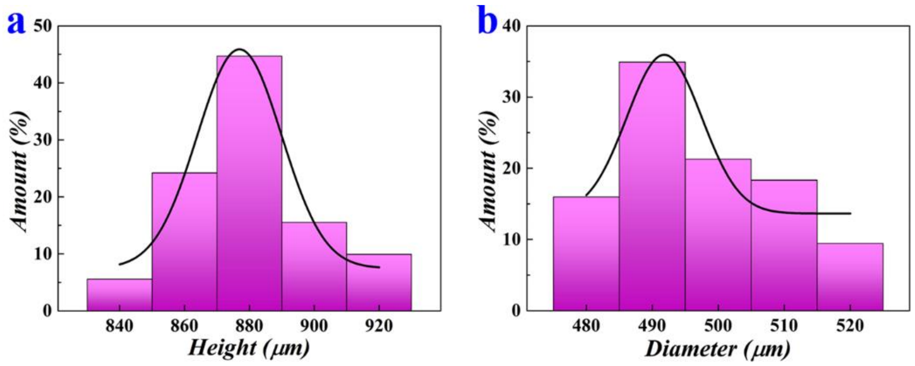

Figure S1. Uniformity analysis of the heights (**a**) and diameters (**b**) of tips of ice microneedles (n=160).

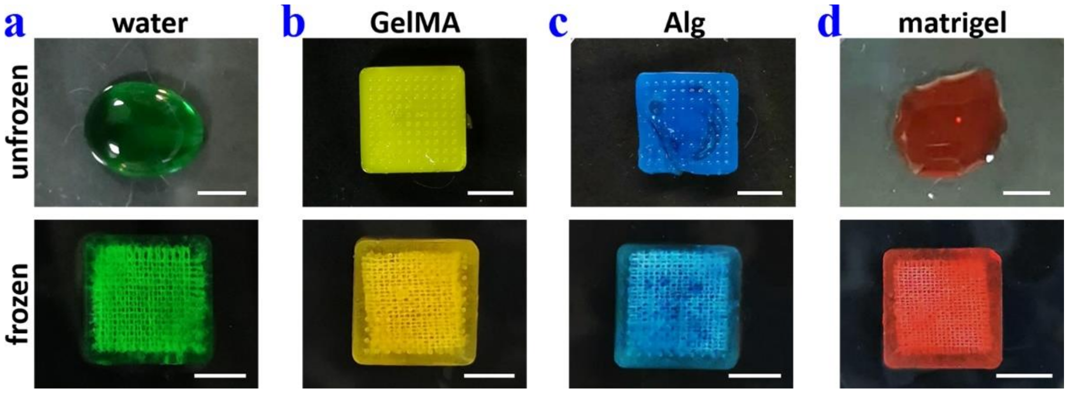

Figure S2. Characterization of unfrozen microneedles and ice microneedles made of water (**a**), GelMA (**b**), Alg (**c**), and Matrigel (**d**). All scale bars: 0.5 cm.

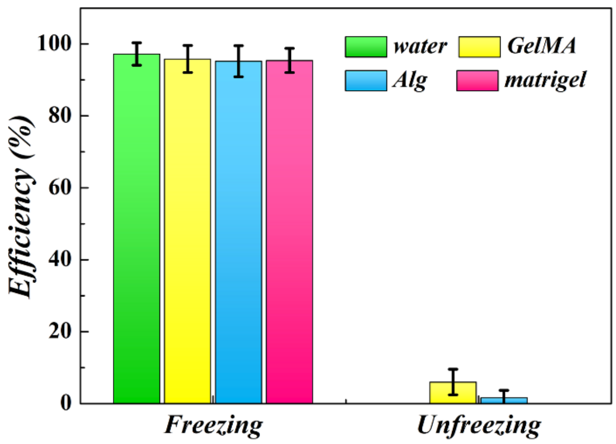

Figure S3. Penetration efficiency of ice microneedles and unfrozen microneedles on agarose (n=5 for each group).

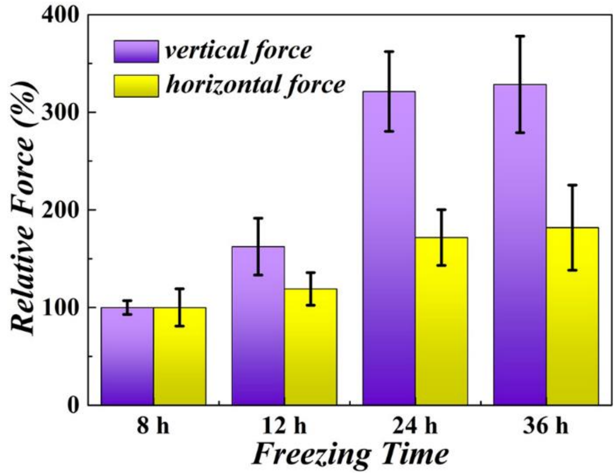

Figure S4. Influence of freezing time on the mechanical strengths of ice microneedles (n=4 for each group).

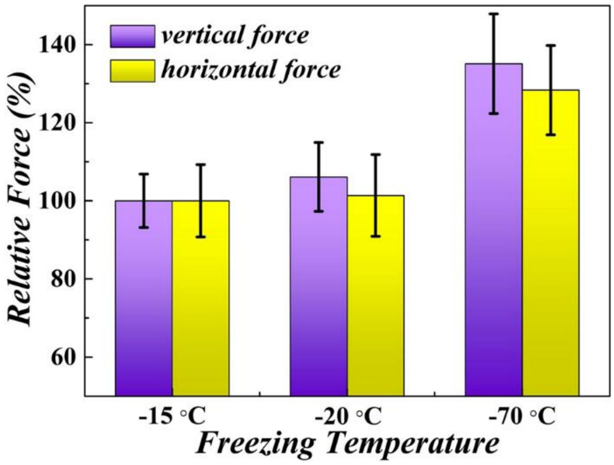

Figure S5. Influence of freezing temperature on the mechanical strengths of ice microneedles (n=4 for each group).

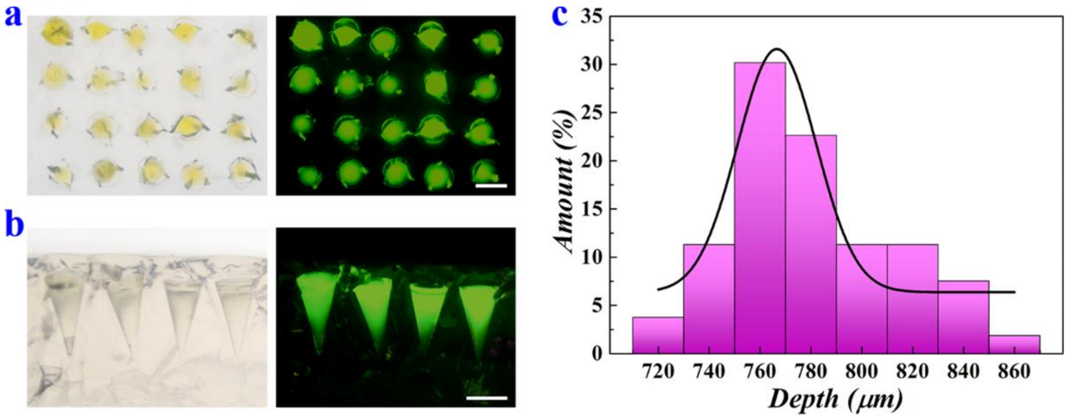

Figure S6. Penetration and separation abilities of ice microneedles in agarose. (**a**) Bright field and fluorescence field images of ice microneedle tips inside the agarose from the top view. (**b**) Bright field and fluorescence field images from the side view. (**c**) Corresponding statistics of the penetration depths of the ice microneedles (n=100). Both scale bars: 500 μm.

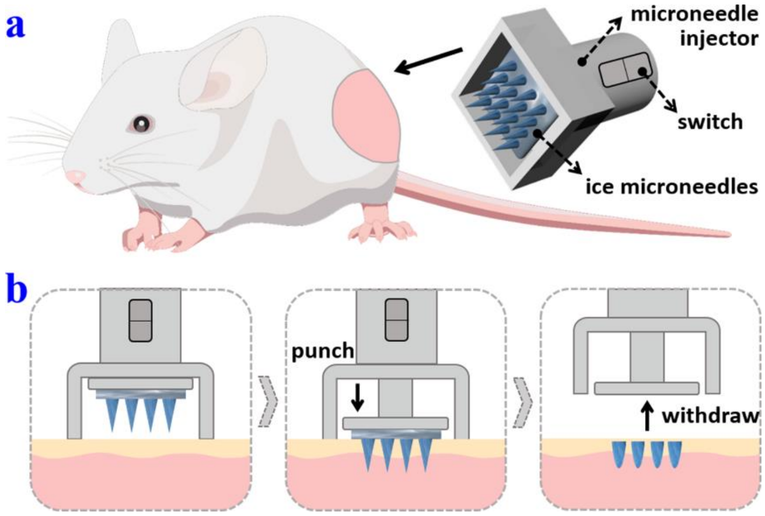

Figure S7. Schematic illustrations of applying ice microneedles via a microneedle injector. (**a**) Composition of the microneedle injector and its application on the mouse. (**b**) The operation procedures.

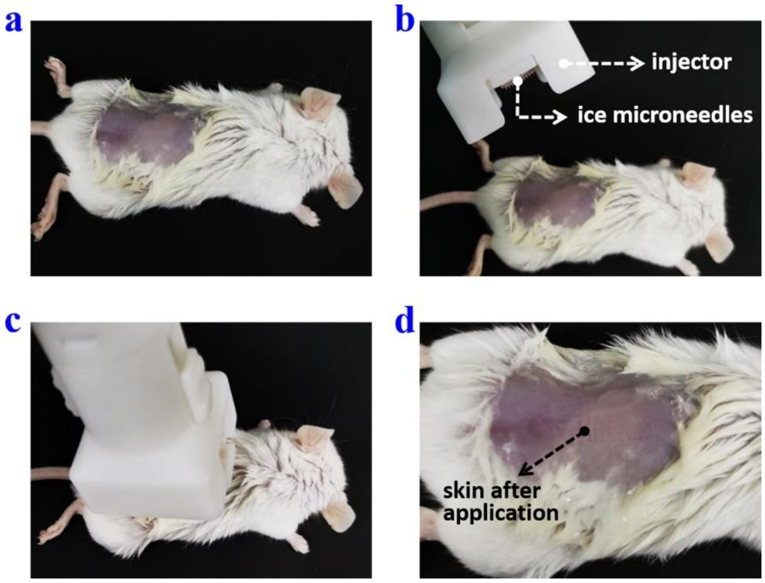

Figure S8. Digital images of applying ice microneedles to the mouse by the microneedle injector. (**a**) The anesthetized mouse with a naked back. (**b**) The microneedle injector approaches the mouse. (**c**) The microneedle injector switches on and the ice microneedles penetrate the skin. (**d**) Removal of the injector and the skin after application.

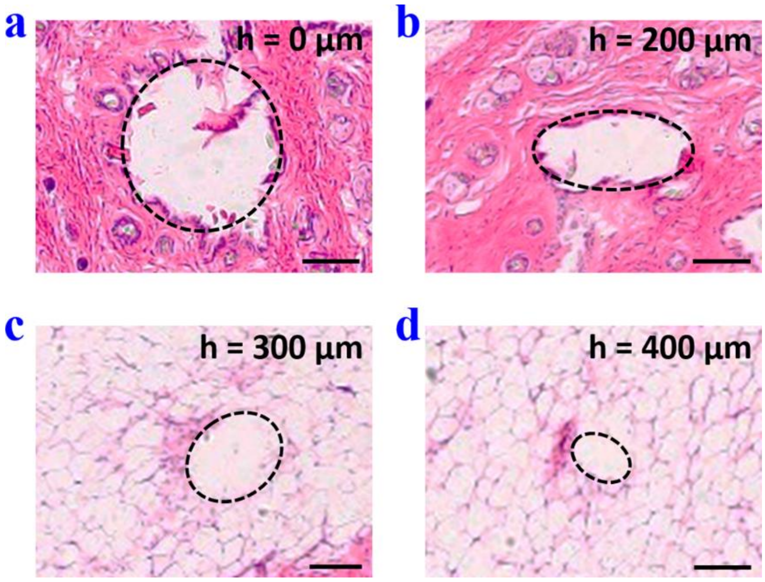

Figure S9. H&E staining images of cross sections of the ice-microneedle-applied mouse dorsal skin. The images differ in z axis. Scale bar:100μm.

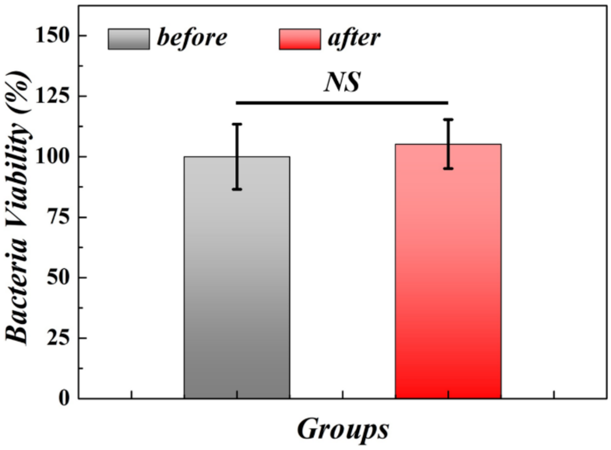

Figure S10. Quantitative analysis of bacteria viability in hydrogel blocks before and after freezing (n=4 for each group; Student's t-test was conducted for comparsion; NS: nonsignificant).

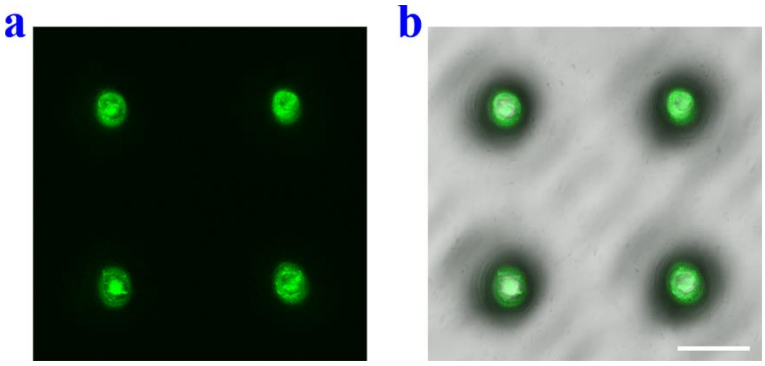

Figure S11. Confocal microscopy images of *B. subtilis* inside ice microneedles. (**a**) The fluorescence image. (**b**) The superposed image of the bright field and the fluorescence image. Scale bar: 500 μm.

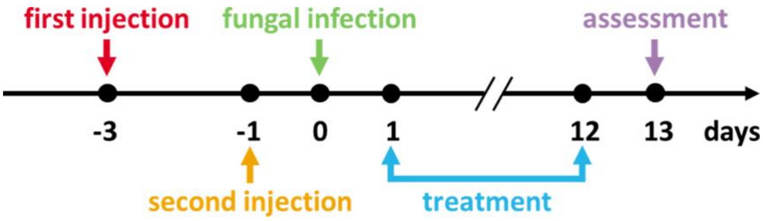

Figure S12. The flow diagram of establishment and treatment of the cutaneous fungal infection mouse model.

B. subtilis (n=6).

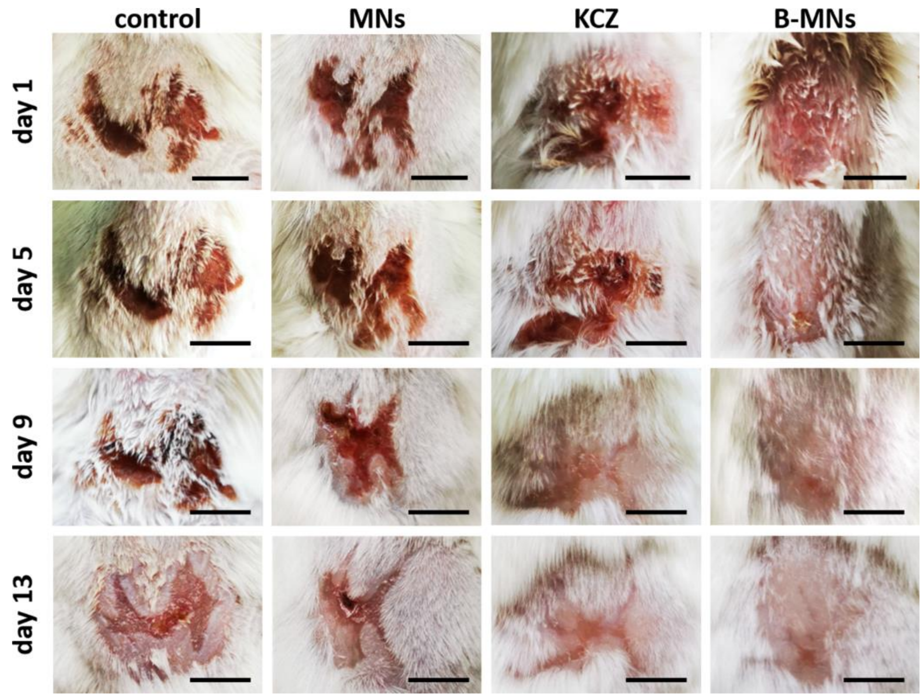

Figure S13. Images of the mouse back skins from the control group, the MNs group, the KCZ group, and the B-MNs group on day 1, day 5, day 9, and day 13. All scale bars: 0.5 cm.

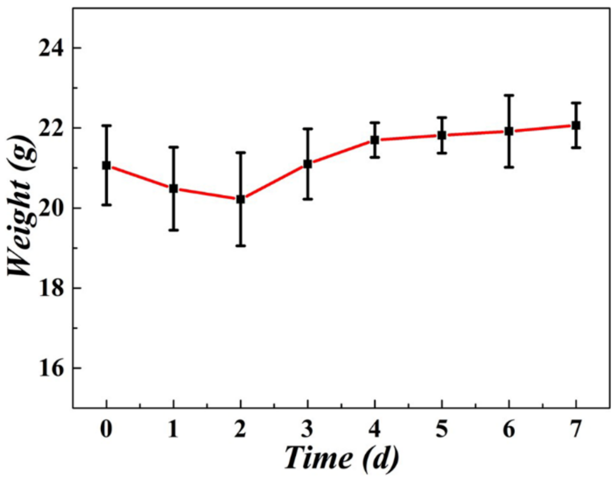

Figure S14. Body weight changes of mice subcutaneously injected with high concentration of *B. subtilis* (n=6).

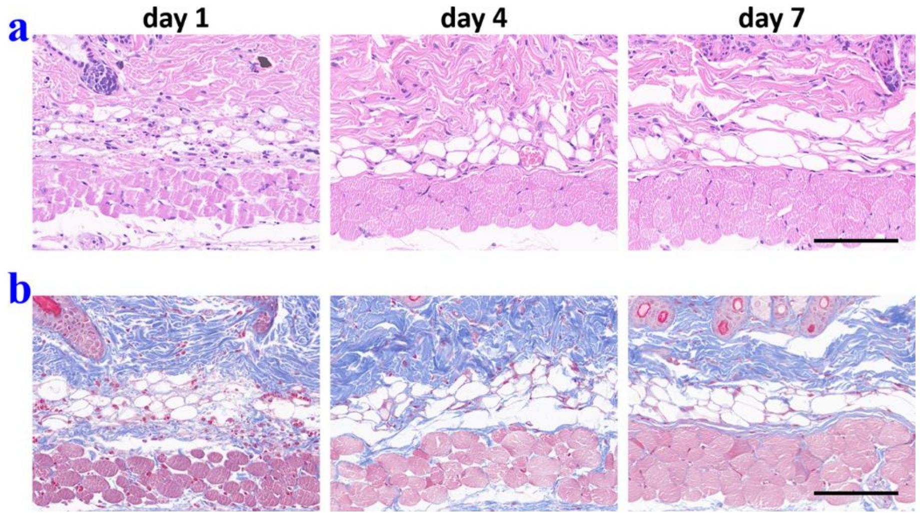

Figure S15. H&E staining (**a**) and Masson staining (**b**) of the mouse back skins on day 1, day 4, and day 7 after bacteria injection. Scale bars: 100 μm.
